# Views Toward Pharmacogenomic Testing Among Patients With Cancer

**DOI:** 10.1001/jamanetworkopen.2025.26714

**Published:** 2025-08-08

**Authors:** Sharon P. Shriver, Sarah Long, Mark E. Fleury

**Affiliations:** 1 American Cancer Society Cancer Action Network

## Abstract

This survey study assesses views and understanding of pharmacogenomic testing among patients with cancer and cancer survivors.

## Introduction

Pharmacogenomic (PGx) testing identifies genetic variants that govern individual differences in drug metabolism, including variants that can increase one’s risk of adverse events, death, or inefficacy in response to certain medications. Pretreatment PGx testing has been shown to effectively inform treatment decisions and improve patient outcomes in oncology, and clinician willingness to integrate PGx into clinical practice is increasing.^1,2^ This survey study assessed understanding of and attitudes of patients with cancer toward PGx testing as a step toward effective implementation of PGx.^3^

## Methods

The American Cancer Society Cancer Action Network’s Survivor Views^4^ web-based survey was fielded June 26 to July 19, 2023, to patients and survivors nationwide who were diagnosed with or treated for cancer in the last 7 years. Participants reviewed electronic informed consent information, and the study was determined exempt by the Morehouse School of Medicine institutional review board as a benign behavioral intervention. Differences reported between groups were tested for statistical significance at a 95% CI and a 2-sided *P* < .05 using SPSS version 30 (IBM). More details on recruitment strategy and methods are in the eMethods in Supplement 1. Reporting follows the AAPOR guideline.

## Results

Of the 1155 respondents (267 aged 45-54 years [23%]), 1013 (88%) were female, 94 (8%) were Black or African American, 30 (3%) were multiracial, 996 (86%) were White, 1083 (94%) were non-Hispanic, and 760 (66%) had breast cancer. When asked how concerned they would be about a clinician administering a drug for which 1% of patients would experience a severe reaction, 647 respondents (56%) reported they would be very or somewhat concerned about taking this drug, while 462 (40%) reported they would be not very or not at all concerned (Table).

**Table.** Concerns With Taking a Drug With a 1% Severe Toxicity Rate, Before and After Learning About PGx Testing

Level of concern	Participants, No. (%) (N = 1155)		*P* value^a^
	Before learning about PGx testing	After learning about PGx testing	
Concerned			
Total	647 (56)	805 (70)	<.001
Very concerned	152 (13)	360 (31)	<.001
Somewhat concerned	495 (43)	445 (39)	.03
Unconcerned			
Total	462 (40)	281 (24)	<.001
Not too concerned	381 (33)	228 (20)	<.001
Not at all concerned	81 (7)	53 (4)	.01
Not sure	46 (4)	69 (6)	.03

Abbreviation: PGx, pharmacogenomic.

^a^ Two-tailed *z* test for proportions.

The survey then provided this definition of PGx:

“Each person has a slightly different inherited genetic makeup that can cause different people to have different responses to the same drug. These differences may impact the effectiveness of a drug or the side effects a person may experience. (Note: this is different from biomarker testing of a tumor to help determine which drug may be used).”

Based on this definition, 480 respondents (42%) indicated presurvey awareness that PGx testing was possible, and 511 (44%) said they were not previously aware. Among those previously aware of PGx testing, 216 (45%) heard about it from a health care practitioner. One-third of respondents (158 respondents [33%]) learned about PGx testing through their own research, such as online, books, articles, or school. There were 39 respondents (8%) who discovered PGx testing through friends or family, while 20 (4%) read about it in medication information.

Finally, respondents were asked a modified version of the initial survey item: how concerned would they be about a clinician administering a drug to which 1% of patients have a severe reaction, without first testing the respondent’s potential for a severe reaction? A combined 70% (805 respondents) reported they would be concerned about taking the drug without PGx testing, while 24% (281 respondents) reported not being concerned (Table).

## Discussion

A core tenet of patient-focused drug development is “…that stakeholders engage with patients and other appropriate subject matter experts… to evaluate…perspectives on treatment benefits and risks.”^5^ In light of recent debates about the benefit-risk balance of pretreatment PGx testing as standard of care, the findings of this survey study add critical patient perspective. The majority of respondents were not aware of PGx testing, and most of those that were aware learned about it from sources other than their clinician. Regardless of prior knowledge, most were concerned about receiving a drug with a low frequency of serious adverse events, and concern increased with knowledge that a PGx test was available but not administered. Label information and clinician guidelines should ensure patient information needs are met. Limitations included the survey respondents being a nonprobabilistic panel not stratified by treatment type and PGx testing not being relevant to all treatment regimens, which may have affected patient awareness of PGx testing.

## Notes

Supplement 1. **eMethods.** Survey Questions and Methodology

Supplement 2.
Data Sharing Statement
