# Supplementary material for: Views Toward Pharmacogenomic Testing Among Patients With Cancer
Source: JAMA Netw Open. 2025 Aug 8;8(8):e2526714. doi: 10.1001/jamanetworkopen.2025.26714 (PMC12334949; doi:10.1001/jamanetworkopen.2025.26714)
Supplement: Supplement 1. — eMethods. Survey Questions and Methodology [file jamanetwopen-e2526714-s001.pdf]

Supplemental Online Content

Shriver SP, Long S, Fleury ME. Views toward pharmacogenomic testing among patients with cancer. *JAMA Netw. Open*. 2025;8(8):e2526714. doi:10.1001/jamanetworkopen.2025.26714

eMethods. Survey Questions and Methodology

This supplemental material has been provided by the authors to give readers additional information about their work.

© 2025 Shriver SP et al. *JAMA Network Open*.

eMethods. Survey Questions and Methodology

This web-based survey was fielded June 26-July 19, 2023, among 1,155 cancer patients and survivors nationwide who have been diagnosed with or treated for cancer in the last seven years. More detailed methodology is described below.

The following survey questions were accompanied by additional questions on other topics as part of an omnibus survey.

Full Question Text

If you were prescribed a drug for your cancer that a small number of patients (1%) have a severe reaction to, how concerned would you be about taking the drug yourself?

Very concerned
Somewhat concerned
I'm not sure
Not too concerned
Not at all concerned

Each person has a slightly different inherited genetic makeup that can cause different people to have different responses to the same drug. These differences may impact the effectiveness of a drug or the side effects a person may experience. Before this survey, were you aware that it is possible to test a patient's overall genetic makeup to understand potential differences in how they may respond to a drug? (Note: this is different from biomarker testing of a tumor to help determine which drug may be used)

Yes
No
I'm not sure

[IF YES ABOVE:] How did you learn about the potential for your genes to affect your response to certain drugs, also known as PGx or pharmacogenomics?

My provider (doctor, oncologist, nurse, pharmacist) told me about it
Personal research (internet, books, articles, school, etc.)
Friends, family, or other patients told me about it
Read about it in information about the drugs I took
I'm not sure/don't recall

If you were prescribed a drug that a small number of patients (1%) have a severe reaction to, but for which a PGx test might be able to identify your risk of a severe reaction in advance, how concerned would you be about your provider administering the drug without first testing your potential for a severe reaction beforehand?

Very concerned
Somewhat concerned
I'm not sure

© 2025 Shriver SP et al. *JAMA Network Open*.

Not too concerned
Not at all concerned

Methodology

ACS CAN's *Survivor Views* research initiative was designed to support the organization's efforts to end suffering and death from cancer through public policy advocacy. The survey population is composed of individuals who meet the following criteria:

- Diagnosed with and/or treated for cancer within the last seven years
- Over the age of 18 (parents of childhood cancer survivors were invited to participate on behalf of their minor children)
- Reside in the US or US territories

Potential Survivor Views participants were invited to participate through email invitations, social media promotion, and partner group outreach. Those who agreed to participate after reviewing the informed consent information completed a brief survey including demographic and cancer history information to inform analysis as well as topical questions as discussed in this document. The data were collected between June 26-July 19, 2023. A total of 1,155 cohort participants responded to the survey. Differences reported between groups are tested for statistical significance at a 95% confidence interval.

AAPOR Transparency Initiative Disclosure Elements (revised April 2021)

These items correspond to the first part of Section III.A of AAPOR's Code of Professional Ethics and Practices, which was revised in April 2021.

1. **Data Collection Strategy:** The research consists of an online survey of ACS CAN's Survivor Views research cohort.

2. **Who Sponsored the Research and Who Conducted It:** This research was conducted as part of ACS CAN's ongoing Survivor Views research project, and administered by project manager Sarah Long. Ms. Long's salary is funded in part by institutional grants to ACS CAN by Bristol Myers Squibb, however no funder or sponsor had any role in the development, design, administration, analysis, or reporting of the research.

3. **Measurement Tools/Instruments.** The measurement tool is a web-based survey consisting of four closed-ended questions related to concerns about adverse pharmacological effects and PGx testing (listed above). In addition, Survivor Views participants answer a series of screening questions to ensure they are over age 18, residents of the United States, and have been treated or diagnosed with cancer within the past seven years (full text available upon request). They also answer a series of twelve demographic questions (listed below).

4. **Population Under Study.** The Survivor Views research cohort is comprised of US adults who have been treated for or diagnosed with cancer within the past seven years. The study protocols submitted for Institutional Review Board exempt status specify that only adult participants over age 18 living in the US will be asked to sign the Survivor Views Informed Consent agreement. The

© 2025 Shriver SP et al. *JAMA Network Open*.

decision to include only patients treated or diagnosed within the past seven years helps to ensure the cohort reflects recent conditions in cancer treatment and access to care.

5. **Method Used to Generate and Recruit the Sample.** The Survivor Views research cohort is a panel developed and maintained by ACS CAN. Participants are recruited from many sources including the American Cancer Society's database of over 1.86 million constituents who have interacted with the American Cancer Society and the resources it provides to cancer patients and their families within the past five years. Participants are randomly selected from this database and invited to participate in the cohort. To ensure broad representation, Survivor Views also periodically shares information about joining the Survivor Views cohort through partner organizations and patient support groups interacting with cancer patients and survivors through digital and in person outreach. Those who agree to join the cohort after reviewing the informed consent information are offered to participate in up to six web-based surveys per year. Occasionally, Survivor Views cohort participants are offered incentives such as $25 gift cards in appreciation for their time, however no incentives were specifically offered in relation to this survey. Internet access and basic computer skills are a limitation of the sampled population.

6. **Method(s) and Mode(s) of Data Collection.** This web-based survey was conducted online via email invitations to the cohort.

7. **Dates of Data Collection.** June 26-July 19, 2023.

8. **Sample Sizes** A total of 1,155 participants responded to the online survey. The analysis does not rely on measures of population projectability such as theoretical margin of sampling error, rather the analysis is limited to the difference in response to two survey variables within the survey instrument before and after another response variable.

9. **How the Data Were Weighted.** Unweighted.

10. **How the Data Were Processed and Procedures to Ensure Data Quality.** Survivor Views surveys include validity checks designed to mitigate the risks associated with web-based data collection. The initial recruitment stage in which participants sign an Informed Consent statement requires question logic and consistency checks. Any speeders and straight-line respondents are removed from the cohort, and open-ended questions and IP addresses are used to identify potential bots.

11. **Limitations of the Design and Data Collection.** The manuscript includes a general statement acknowledging limitations specific to this study. Another limitation of the study design and data collection is the exclusion of cancer patients and survivors without access to a broadband connected device.

Participant Demographic Questions

Patient Age

0-17 ---

18-24 ---

© 2025 Shriver SP et al. *JAMA Network Open*.

25-34 ---

35-44 ---

45-54 ---

55-64 ---

65-74---

75 or older ---

About how long ago did you receive your last cancer treatment?

I am currently in treatment ---------------------------

I completed treatment less than a year ago --------

1 year ago to less than three years ago --------------

3 years ago to less than 5 years ago -----------------

5 years ago to less than 10 years ago ----------------

10 years ago to 20 years ago -------------------------

More than 20 years ago--------------------------------

I have not been treated for cancer -------------------

About how long ago was the diagnosis made of the cancer that you have most recently been treated for?

I was diagnosed within the past six months --------

More than six months to less than a year ago

1 year ago to less than three years ago --------------

3 years ago to less than 5 years ago -----------------

5 years ago to less than 10 years ago ----------------

10 years ago to 20 years ago -------------------------

More than 20 years ago--------------------------------

© 2025 Shriver SP et al. *JAMA Network Open*.

What type of cancer have you been diagnosed with? (select all that apply)

Breast cancer ---

Skin cancer, melanoma & non-melanoma ---------

Cervical, ovarian, or endometrial cancer -----------

Blood cancer (Lymphoma, Leukemia) --------------

Colorectal cancer --------------------------------------

Bladder or renal (kidney) cancer ---------------------

Prostate cancer ---

Lung cancer ---

Bone cancer ---

Oral, pharyngeal, or head and neck cancer --------

Liver cancer ---

Pancreatic cancer --------------------------------------

Stomach cancer --

Other (specify) --

© 2025 Shriver SP et al. *JAMA Network Open*.

What is your primary source of health insurance or health care coverage?

Employer-provided insurance------------------------

Medicare (including Medicare Advantage or other supplemental Medicare plans)---------------------

Privately purchased health insurance – for example, through the marketplace----------------

Medicaid **[INSERT STATE-SPECIFIC LABEL]** ------

Military health care (TRICARE/VA/CHAMP-VA)-----

Other (specify) --

No coverage of any type-------------------------------

Indian Health Service----------------------------------

SCHIP (CHIP/Children's Health Insurance Program)--

I don’t know --

Are you of Hispanic, Latino/a, or Spanish origin?

No ---

Yes ---

What is your race? (Select all that apply)

White --

Black or African American -----------------------------

Multiracial/Two or more races ------------------------

American Indian or Alaska Native --------------------

Asian/Pacific Islander ---------------------------------

Other --

How do you describe yourself?

© 2025 Shriver SP et al. *JAMA Network Open*.

Female ---

Male--

Gender non-binary-------------------------------------

Which of the following best describes your sexual orientation?

Heterosexual or straight -------------------------------

Asexual--

Lesbian--

Bisexual ---

Gay---

Pansexual---

Queer---

Other ---

I prefer not to answer ----------------------------------

Thinking about all family members living in your household, what is your combined annual income from all sources (before taxes)?

$35,000 or less--

$35,001 to $70,000 ------------------------------------

$70,001 to $125,000-----------------------------------

$125,001 or more --------------------------------------

I prefer not to answer ----------------------------------

What is the highest grade or level of schooling you completed?

Less than high school degree ------------------------

© 2025 Shriver SP et al. *JAMA Network Open*.

High school degree or equivalent (GED) ------------

Post high school training other than college
(vocational or technical)-----------------------------

Some college--

Associate degree ---------------------------------------

Bachelor's degree --------------------------------------

Post-graduate/Advanced degree ---------------------

Which of the following best describes the area where you live?

Suburban --

Rural ---

Urban --

© 2025 Shriver SP et al. *JAMA Network Open*.
