# Supplementary material for: Views Toward Pharmacogenomic Testing Among Patients With Cancer
Source: JAMA Netw Open. 2025 Aug 8;8(8):e2526714. doi: 10.1001/jamanetworkopen.2025.26714 (PMC12334949; doi:10.1001/jamanetworkopen.2025.26714)
Supplement: Supplement 2. — Data Sharing Statement [file jamanetwopen-e2526714-s002.pdf]

Data Sharing Statement

Data
Data available: Yes
Data types: Data (not involving human participants)
How to access data: Survey data will be made available upon request to Sharon Shriver at sharon.shriver@cancer.org
When available: With publication

Supporting Documents
Document types: None

Additional Information
Who can access the data: Survey data will be made available to researchers or others upon approval.
Types of analyses: Survey data may be used for research, education, or policy purposes.
Mechanisms of data availability: Survey data will be made available to researchers or others upon approval of a proposal for use.
